# The oral contraceptive cycle and its influences on maximal and submaximal endurance parameters in elite handball players

**DOI:** 10.3389/fphys.2024.1305895

**Published:** 2024-03-27

**Authors:** Astrid Mathy, Barbara Wessner, Patricia Haider, Harald Tschan, Christoph Triska

**Affiliations:** ^1^ Leistungssport Austria, Brunn am Gebirge, Austria; ^2^ Department of Sport and Human Movement Science, Centre for Sport Science and University Sports, University of Vienna, Vienna, Austria; ^3^ Research Platform Active Ageing, University of Vienna, Vienna, Austria

**Keywords:** withdrawal bleeding, athletes, hormonal contraceptive, female endurance performance, endurance, hormonal cycle, monophasic oral contraceptive pill

## Abstract

The usage of the oral contraceptive pill is widespread among athletes of various levels. However, there is limited knowledge on how the intake of the pill alters the submaximal and maximal endurance parameters between the oral contraceptive phases. Therefore, the aim of this study was to examine potential differences between the pill intake and withdrawal phase on endurance-related parameters in first-division handball players. In total, 15 female team handball players performed two graded exercise tests until volitional exhaustion on a motorized treadmill. Tests were performed during the pill intake (days 16–17) and withdrawal phase (day 2–3). Throughout the test, respiratory gases were measured breath-by-breath, and the heart rate was measured continuously. Before and after the graded exercise test, blood samples were obtained in order to assess the blood lactate concentration. Before each test, venous blood samples were taken to determine endogenous sex hormone levels. Ventilatory parameters ( $\dot{V}$ O_2_, $\dot{V}$ CO_2_, and $\dot{V}$ E, and respiratory equivalents for $\dot{V}$ O_2_ and $\dot{V}$ CO_2_) were measured, and the oxidation of fat and carbohydrates was calculated. A paired-sample *t*-test was used to assess differences between the two time points, and the significance was accepted as *p* < 0.050. Significant differences with lower values during the consumption phase were found for absolute (mean difference ± SD: 88 ± 131 mL∙min^−1^; *p* = 0.021) and relative $\dot{V}$ O_2peak_ (mean difference ± SD: 1 ± 2 mL∙min^−1^∙kg^−1^; *p* = 0.012). Higher values during the consumption phase were found for submaximal respiratory equivalents for $\dot{V}$ O_2_ (mean difference ± SD: −1.1 ± 1.7; *p* = 0.028) and $\dot{V}$ CO_2_ (mean difference ± SD: −0.9 ± 1.5; *p* = 0.032). No differences were found for all other parameters, including differences for endogenous sex hormones (*p* > 0.050). The results of the current study suggest only marginal and physiologically insignificant differences in endurance-related parameters between oral contraceptive phases.

## Introduction

The length of the eumenorrheic menstrual cycle and, consequently, the day of onset of bleeding are, in some athletes, more consistent than in others. According to a recent survey, about half of adolescent athletes reported having irregular menstrual cycles (Rosen et al., 2020), and especially in endurance sports, menstrual irregularities are widely spread across athletes (Rosen et al., 2020). The chance of having a regular menstrual cycle and controlling its timing is a desire of numerous women, particularly competitive athletes (Elliott-Sale et al., 2020).

An often-used oral contraceptive pill (OCP) type is the combined monophasic OCP, which includes exogenous estrogen and exogenous progesterone (also progestogen). It is used for 21 days of consumption (CONS), followed by 7 days of withdrawal (WITH) or the intake of placebo pills. The intake of the exogenous hormones results in endogenous hormones like estradiol (E2), progesterone (prog), and testosterone (testo) remaining at low or stable concentrations or might increase in WITH when bleeding occurs (McNulty et al., 2020). The hormone profile across an oral contraceptive cycle (OCC) is not similar to the hormone profile of regular menstruation (McNulty et al., 2020).

The OCPs are not only used for contraceptive reasons but also for controlling the timing and duration of bleeding as well as to skip bleeding. Furthermore, the intake of OCP reduces symptoms of dysmenorrhea (e.g., cramps, pain, and headache); moreover, it diminishes menorrhagia (McNulty et al., 2020) and facilitates training planning as well as competition scheduling. Therefore, it is not surprising that the usage of OCPs in elite sports is widely spread. Recent data show that approximately a third of adolescent athletes (Rosen et al., 2020) and half of female adult athletes are using OCPs (Martin et al., 2018). In this context, 70% of female elite athletes reported already using hormonal contraceptives (Martin et al., 2018).

It is not fully understood whether the initiation of OCP intake might have a negative influence on physical performance (McNulty et al., 2020), and even variations within the OCC have been discussed lately. In terms of strength performance, most studies found no differences within the OCC, with constant and stable strength performances (Elliott et al., 2005; Ekenros et al., 2013; Elliott-Sale et al., 2020; McNulty et al., 2020; Reif et al., 2021). When it comes to endurance performance, recent results are controversial.

On the one hand, no differences through OCC phases for endurance parameters have been detected (Grucza et al., 1993; Vaiksaar et al., 2011b; Jurimae et al., 2011; Joyce et al., 2013; Gordon et al., 2018). On the other hand, previous literature found increased minute ventilation ( $\dot{V}$ E) during OCP intake compared to WITH (Barba-Moreno et al., 2019). During OCP intake, decreased submaximal oxygen uptake ( $\dot{V}$ O_2_) and enhanced running economy have been found, suggesting a positive influence of OCP intake on physiological and biomechanical factors in running (Giacomoni and Falgairette, 2000). In another study, [blood lactate] (BLa^−^) and rating of perceived exertion, as well as breathing frequency, were higher during OCP intake; however, it is concluded that there is no need to be concerned about or manipulate OCC to optimize endurance performance for competitions (Rechichi et al., 2009). This is in accordance with another study reporting differences in ventilation and interpreting them as not relevant for cycling competition (Vaiksaar et al., 2011a).

A number of studies have been conducted on maximal and submaximal endurance-related parameters, but the results are contradictory. The inconsistency of previous research might be explained by the heterogeneous training status of the study participants and also by different types of sports and disciplines. The present study aims to minimize the lack of data on consistently well-trained team sport athletes in order to facilitate their transfer into the practice of (elite) sports. According to a recent survey on elite athletes, more than a third of female elite athletes use OCPs (Martin et al., 2018), and the monophasic combined pill is the most commonly used OCP in Europe, according to our experience. It is, however, of great interest to analyze the effects of the intake on submaximal and maximal endurance performance parameters. It is hypothesized that endurance performance-related parameters are enhanced in WITH due to a higher level of endogenous E2.

## Materials and methods

### Participants

The sample consisted of 15 female team sport athletes playing in the first Austrian handball league and training at least three times per week (age: 22.9 ± 3.2 years, body mass: 66.9 ± 8.6 kg, and body stature: 1.69 ± 0.08 m). Furthermore, inclusion criteria were a training history of at least 3 years and the use of a commercially available low- to middle-dose monophasic OCP (0.020–0.035 mg ethinylestradiol combined with 0.10–2.00 mg gestodene). Participants used OCPs, which were characterized by 21 days of OCP intake and an additional 7 days of WITH when no-pill or placebo pills were taken (introducing withdrawal bleeding). Moreover, participants were included in the study only when they used OCP for more than half a year and according to the instructions. An additional calculation of subgroups has been performed, including only participants with lower hormone levels in CONS than in WITH.

### Study design

The present cross-sectional study evaluates endurance-related parameters [i.e., $\dot{V}$ O_2_, $\dot{V}$ CO_2_, $\dot{V}$ E, RE, substrate metabolism, (BLa^−^), and heart rate] across a single OCC of female team sport athletes. Rechichi et al. (2009) reported that the intake of OCPs is associated with a more stable concentration of sex hormones. Consequently, the evaluation of a single OCC seemed feasible for this research since endogenous hormones stay low during chronic OCP intake and the dosage of exogenous hormone intake and the length of an OCC are predictable. Participants were reported to the laboratory twice to analyze aerobic endurance performance in two different phases of the OC. First, in the WITH phase, which lasts for 7 days and includes menses (testing days on days 2 or 3 of WITH), WITH has been defined as the reference state for comparisons. Second, during the CONS phase, which lasts for 21 days, athletes were tested on days 16 or 17 of the OCP cycle, when OCP consumption had been re-initiated for 9 or 10 days. To avoid a potential bias caused by the order of testing days, visits in WITH and CONS were in different orders. During both visits, participants performed a graded exercise test (GXT) until volitional exhaustion to evaluate potential differences in aerobic endurance performance. Athletes were in a fed and hydrated state when they arrived at the laboratory and were instructed to replicate their food intake the day prior, from the first to the second test. Subjects were required to avoid intense exercise and alcohol for 24 h prior to testing. In addition, caffeine and sports drink intake were not allowed in the last 3 h prior to the tests. The tests were performed in a humidity- and air-condition-controlled laboratory at 20°C–22°C and between 45% and 55% humidity. To avoid potential effects of the diurnal rhythm, tests were performed at the same time of the day (±1 h) for each person. Participants were fully informed of all testing procedures and risks of this study and provided written informed consent. The University of Vienna´s Ethics Committee (#00435) approved all procedures of the study, which conformed to the principles of the World Medical Association’s Declaration of Helsinki (2013).

### Measures

#### Pre-test measures and warm-up

Using a commercially available stadiometer and scale prior to each test, body stature and body mass were measured; the latter was to examine potential differences in time points across OCC phases. Subsequently, participants executed a standardized warm-up program with a predefined workload of 0.75 W/kg body mass for 10 min on a stationary ergometer (Racer 9, Kettler Freizeit GmbH, Ense-Parsit, Germany).

#### Graded exercise test

To obtain relevant endurance-related parameters, a GXT on a motorized treadmill (Saturn, h/p/cosmos, Traunstein, Germany) was performed during WITH and CONS. After baseline walking at 1.39 m∙s^−1^ for 3 min (which was used for baseline $\dot{V}$ O_2_ measures), athletes commenced running at 1.67 m∙s^−1^ with an increment of 0.14 m∙s^−1^ every minute until volitional exhaustion, despite strong verbal encouragement by the investigators. The treadmill incline was set to 1%. During the test, respiratory gases were measured breath-by-breath using a mobile gas analyzer (MetaMax3B-R2, Cortex Biophysik GmbH, Leipzig, Germany). Before each test flow, volume was calibrated using a 3-L syringe, and the gas analyzer was calibrated according to the recommendations of the manufacturer using known gases (15% O_2_ and 5% CO_2_, Cortex Medical GmbH).

#### Submaximal measures and determination of thresholds

Submaximal $\dot{V}$ O_2_, respiratory exchange ratio (i.e., the ratio of $\dot{V}$ CO_2_ and $\dot{V}$ O_2_; RER), $\dot{V}$ E, as well as ventilatory equivalents for O_2_ and carbon dioxide (CO_2_) (i.e., $\dot{V}$ E/ $\dot{V}$ O_2_ and $\dot{V}$ E/ $\dot{V}$ CO_2_), and submaximal heart rate (HR) at 2.50 m∙s^−1^ work stage were analyzed during the last 30 s and 5 s, respectively. Submaximal measurement for $\dot{V}$ O_2_, running economy, and respiratory exchange ratio had to be excluded for one participant as the respiratory exchange ratio was higher than 1.00 during the final 30 s of the 2.50 m∙s^−1^ stage, indicating non-steady-state conditions. Running economy was calculated as oxygen consumption for 1 km at 2.50 m∙s^−1^, normalized to body mass, and expressed as mL∙kg^−1^∙km^−1^.

Ventilatory threshold (VT) 1 was determined using the following criteria: 1) an increase in $\dot{V}$ E/ $\dot{V}$ O_2_ without a simultaneous increase in $\dot{V}$ E/ $\dot{V}$ CO_2_, 2) the first loss of linearity in minute ventilation ( $\dot{V}$ E), and 3) a non-linear increase in $\dot{V}$ CO_2_ (Beaver et al., 1986). VT2 was determined using the following criteria: 1) a secondary increase in $\dot{V}$ E/ $\dot{V}$ O_2_ and $\dot{V}$ E/ $\dot{V}$ CO_2_, 2) changes in the end-tidal respiratory pressure (increase in PetO_2_ and decrease in PetCO_2_), and 3) a secondary increase in $\dot{V}$ E (Wasserman, 1984).

#### Maximal measures

The highest 30-s rolling average measured was accepted as $\dot{V}$ O_2peak_, and the highest speed obtained was considered the maximal aerobic speed (MAS). If the last work rate could not be fully completed, MAS was calculated using Eq. 1 proposed by Kuipers et al. (1985):

MAS=sL+t/60×0.14, (1)

where MAS is the maximal aerobic speed, sL is the speed of the last fully completed work stage (m∙s^−1^), and t is the time of the not fully completed work stage (s). Maximal values were also obtained for $\dot{V}$ E, as well as $\dot{V}$ E/ $\dot{V}$ O_2_ and $\dot{V}$ E/ $\dot{V}$ CO_2_.

#### Carbohydrate and fat oxidation and total energy expenditure

The oxidation of carbohydrate (CHO) and fat was estimated using measures of $\dot{V}$ O_2_ and $\dot{V}$ CO_2_. To calculate substrate oxidation under steady-state conditions, the data of the last 30 s of the 2.50 m∙s^−1^ stage were used (*n* = 14). The following equations, according to Jeukendrup and Wallis (2005), for moderate-to-high-intensity exercise were used in order to calculate CHO and fat oxidation; Eqs 2, 3 were modified in order to convert g∙min^−1^ into J∙s^−1^:

$$\text{CHO oxidation }\left(J∙s^{‐1}\right)=\left(4.210\;x\;\dot{V}{\text{CO}}_{2}\;–\;2.962\;x\;\dot{V}O_{2}\right)\;x\;284.0,$$ (2)

$$\text{Fat oxidation }\left(J∙s^{‐1}\right)=\left(1.695\;x\;\dot{V}O_{2}\;–\;1.701\;x\;\dot{V}{\text{CO}}_{2}\right)\;x\;680.3$$ (3)

Total energy expenditure was estimated as the sum of CHO and fat oxidation, whereas the contribution of protein oxidation was neglected (Jeukendrup and Wallis, 2005).

#### Heart rate

Throughout the GXT, HR was measured continuously using a chest-strapped attached HR sensor (H7, Polar Electro Oy, Kempele, Finland), which was connected via Bluetooth™ to the gas analyzer. HR_max_ was taken as the highest value throughout the GXT. Due to the malfunction of the HR sensor in a single participant, one dataset for HR is missing (*n* = 14).

#### Blood lactate concentration and venous blood samples

Blood lactate concentration was analyzed at baseline, immediately, and 3 min after terminating the GXT. Blood samples (20 µL) were taken from a hyperemic earlobe and diluted immediately in 1,000 µL of glucose solution. Samples were analyzed using an automated lactate analyzer (Biosen S_Line; EKF-diagnostic GmbH, Barleben, Germany). Furthermore, on both test days, venous blood samples were taken to analyze for endogenous hormone levels of prog, testo, and E2. The blood samples were collected in a serum gel tube, and after a 30-min rest, they were centrifuged for 10 min at a relative centrifugal force of 3,500 × g (Rotina 420R, Hettich, Vienna, Austria). Samples were collected and frozen at −40°C until all samples were analyzed in a certified laboratory using Beckman Coulter Access Immunoassays (Beckman Coulter Inc., Brea, CA, United States).

#### Statistical analyses

All data are reported as means ± standard deviations. The normal distribution of data was checked using the procedures of Shapiro–Wilk and an additional visual inspection of boxplots. A paired-sample *t*-test was used to assess the differences between OCC phases (WITH vs. CONS). In cases of violation of normal distribution and for analysis of subgroups, a non-parametric Wilcoxon signed-rank test has been used, and descriptive data are reported as medians and interquartile ranges. The standard error of the mean was calculated as the ratio of the standard deviation of the mean differences and the square root of the number of participants. Statistical analyses were conducted using SPSS software package version 27 (IBM SPSS Statistics, SPSS Inc., Chicago, United States), and graphs were made using GraphPad Prism (v.10.0.1 for Mac, GraphPad Software, Boston, Massachusetts, United States, “www.graphpad.com”). Significance was accepted at an alpha level of *p* < 0.050.

## Results

**FIGURE 1:**
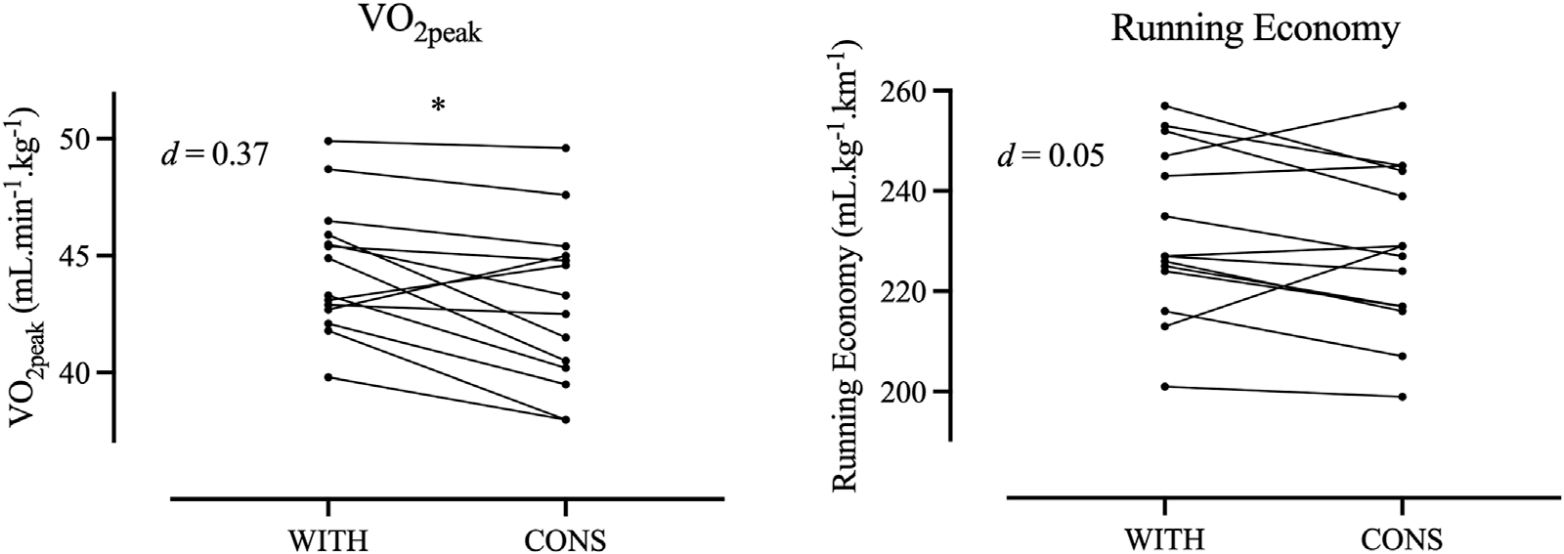
Individual responses for maximal oxygen uptake and running economy during withdrawal phase (WITH) and consumption phase (CONS).

**FIGURE 2:**
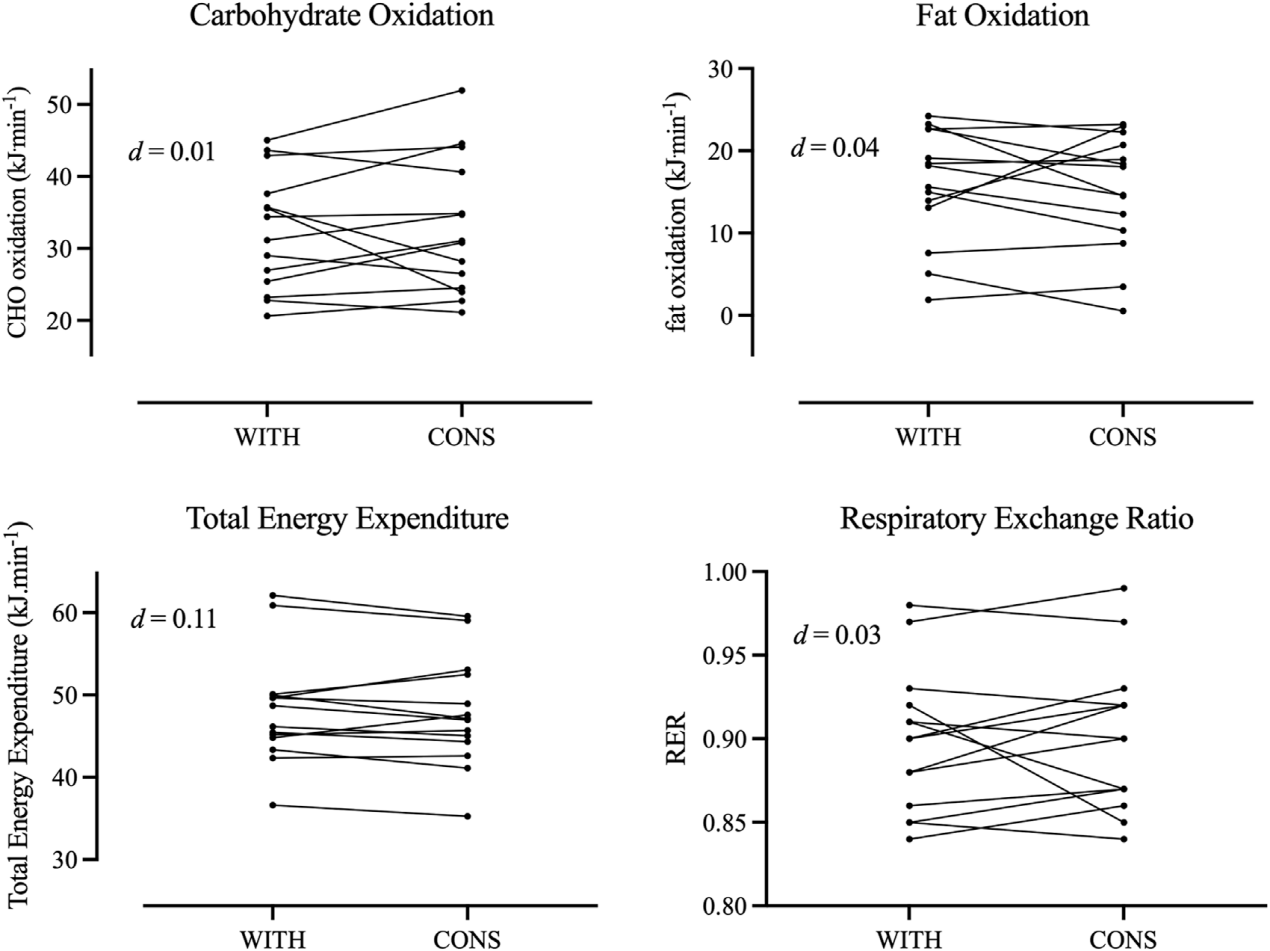
Individual responses for oxidation of carbohydrates and fats as well as for total energy expenditure and respiratory exchange ratio during withdrawal phase (WITH) and consumption phase (CONS).

The mean body mass of the participants (N = 15) was 66.9 ± 8.6 kg, body stature was 1.69 ± 0.08 m, and $\dot{V}$ O_2peak_ was 2,950 ± 422 mL∙min^−1^∙in WITH. The results of the treadmill tests are displayed in Table 1. Individual responses between differences in time points are depicted in Figures 1, 2. Significant differences between WITH and CONS were only revealed for absolute and relative $\dot{V}$ O_2peak_ (*p* = 0.021 and *p* = 0.012, respectively) and submaximal ventilatory equivalents for O_2_ and CO_2_ (*p* = 0.028 and *p* = 0.032, respectively). However, the effect sizes were *trivial* to *small*. All other parameters were not significantly different between WITH and CONS (0.104 < *p* < 0.785).

**TABLE 1:** Overview of comparison between withdrawal and consumption of OCP (WITH–CONS).

	N	WITH (mean ± SD)	CONS (mean ± SD)	Mean differences ± SEM	*p*-value
*Endurance parameter*					
Baseline $\dot{V}$ O_2_ (mL·min^−1^)	15	969 ± 115	936 ± 93	−33 ± 19	0.104
$\dot{V}$ O_2_ at 2.50 m·s^−1^ (mL·min^−1^)	14	2,347 ± 334	2,310 ± 310	−37 ± 22	0.113
RE at 2.50 m·s^−1^ (mL·kg^−1^·km^−1^)	14	232 ± 17	228 ± 16	−4 ± 2	0.128
RER at 2.50 m·s^−1^	15	0.90 ± 0.04	0.90 ± 0.04	0.00 ± 0.01	0.760
Relative $\dot{V}$ O_2peak_ (mL·min^−1^·kg^−1^)	15	44 ± 3	43 ± 4	−1 ± 1	0.012*
Absolute $\dot{V}$ O_2peak_ (mL·min^−1^)	15	2,950 ± 422	2,862 ± 462	−88 ± 34	0.021*
MAS (m·s^−1^)	15	3.51 ± 0.25	3.46 ± 0.30	−0.05 ± 0.03	0.144
VT1 (m·s^−1^)	15	2.10 ± 0.21	2.13 ± 0.24	0.03 ± 0.03	0.374
VT2 (m·s^−1^)	15	2.95 ± 0.25	2.96 ± 0.28	0.02 ± 0.04	0.665
$\dot{V}$ E_max_	15	110 ± 13	106 ± 15	−3 ± 1.5	0.071
$\dot{V}$ E at 2.50 m·s^−1^	15	65 ± 10	67 ± 10	2 ± 1	0.119
$\dot{V}$ E/ $\dot{V}$ O_2_ at exhaustion	15	34 ± 2	34 ± 1	0 ± 0	0.798
$\dot{V}$ E/ $\dot{V}$ O2 at 2.50 m·s^−1^	15	25 ± 1	26 ± 2	1 ± 0	0.028*
$\dot{V}$ E/ $\dot{V}$ CO_2_ at exhaustion	15	31 ± 2	32 ± 2	0 ± 0	0.305
$\dot{V}$ E/ $\dot{V}$ CO_2_ at 2.50 m·s^−1^	15	28 ± 1	29 ± 1	1 ± 0	0.032*
*Energy metabolism*					
Fat oxidation (J·s^−1^)	15	263. ± 116	249 ± 119	−14 ± 22	0.533
CHO oxidation (J·s^−1^)	15	541 ± 135	547 ± 157	7 ± 24	0.785
TTL (J·s^−1^)	15	804 ± 112	796 ± 111	−7 ± 9	0.439
*Metabolic system and heart rate*					
Baseline [BLa^−^] (mmol·L^−1^)	15	1.1 ± 0.3	1.0 ± 0.3	−0.1 ± 0.1	0.228
[BLa^−^]_max_ (mmol·L^−1^)	15	8.5 ± 2.8	8.1 ± 2.4	−0.4 ± 0.4	0.274
HR at 2.50 m·s^−1^ (bpm)	14	167 ± 12	166 ± 14	−1 ± 2	0.521
HR_max_ (bpm)	14	189 ± 9	188 ± 10	−1 ± 1	0.559
*Hormones*					
E2 (pg·mL^−1^)	15	22.5 ± 20.3	15.1 ± 17.8	−7.4 ± 7.4	0.335
Prog (ng·mL^−1^)	15	0.38 ± 0.33	0.34 ± 0.28	−0.04 ± 0.07	0.556
testo (ng·mL^−1^)	15	0.32 ± 0.19	0.28 ± 0.14	−0.04 ± 0.02	0.059
*Anthropometry*					
Body mass (kg)	15	66.9 ± 8.6	67.1 ± 8.8	0.2 ± 0.2	0.433

WITH, withdrawal phase; CONS, consumption phase; SD, standard deviation; SEM, standard error of the mean; $\dot{V}$ O_2_, oxygen uptake; RER, respiratory exchange ratio; [BLa^−^], blood lactate concentration; MAS, maximal aerobic speed; VT, ventilatory threshold; HR, heart rate; CHO, carbohydrate; TTL, total energy expenditure; E2, estradiol; prog, progesterone; testo, testosterone.

### Subgroup analysis

Between OCC phases, a significant difference and *large* effect sizes in E2 (*p* = 0.005), prog (*p* = 0.027), and testo (*p* = 0.005) have been found for the respective subgroups.

In the subgroup that had significantly higher prog levels in WITH than in CONS (*n* = 6), no significant differences were reported in all evaluated parameters (0.075 < *p* < 0.893) except for relative $\dot{V}$ O_2peak_ [WITH: 42.5 mL∙min^−1^∙kg^−1^ (41.2, 46.6); CONS: 40.5 mL∙min^−1^∙kg^−1^ (38.8, 43.8); *p* = 0.028], baseline [BLa^−^] [WITH: 1.2 mmol∙L^−1^ (0.9, 1.4); CONS: 0.9 mmol∙L^−1^ (0.5, 1.2); *p* = 0.044], and [BLa^−^]_max_ [WITH: 7.7 mmol∙L^−1^ (5.8, 12.6); CONS: 6.2 mmol∙L^−1^ (5.8, 11.7); *p* = 0.046].

In the subgroup with significantly lower levels of E2 in CONS than in WITH (*n* = 10), no significant differences were found in all assessed parameters (0.050 < *p* < 0.953).

The analysis of a subgroup with a significant decrease in testo (*n* = 10) from WITH to CONS showed no significant differences in all the assessed parameters (0.066 < *p* < 0.862).

## Discussion

The present study aimed to investigate the differences between OCC phases in endurance-related performance parameters obtained from an incremental running test. The results of the present work suggest no meaningful influence of OCC phases on submaximal systemic responses (e.g., RE, substrate oxidation, and HR). However, maximal oxygen consumption was significantly lower during CONS without an effect on MAS or maximal blood lactate concentration. This might be due to the lower efficiency of the aerobic system during CONS. This is represented by a significantly lower oxygen uptake, which, however, was not translated into a lower maximal running speed. Therefore, the hypothesis that maximal and submaximal endurance performance are reduced during CONS due to a lower level of endogenous E2 was not confirmed.

### Endurance-related parameters

Previous literature is controversial, as some studies did not find significant differences in endurance performance through OCC phases during chronic or short term use of OCP (Grucza et al., 1993; Bryner et al., 1996; Casazza et al., 2002; Vaiksaar et al., 2011b; Jurimae et al., 2011; Joyce et al., 2013; Gordon et al., 2018; Nakamura and Nose-Ogura, 2021), while others found significant differences (e.g., Giacomoni and Falgairette, 2000; Mattu et al., 2020). Across all exercise intensities (i.e., moderate, heavy, and severe exercise intensity domains), lower $\dot{V}$ O_2_ values were found, similar to previous work (Giacomoni and Falgairette, 2000). Furthermore, the present work found significantly lower absolute and relative $\dot{V}$ O_2peak_ values in CONS than in WITH.

It has been previously shown that the chronic use of OCP reduces $\dot{V}$ O_2peak_ (Giacomoni and Falgairette, 2000; Casazza et al., 2002; Suh et al., 2003). Therefore, it might be suggested that even a short-term absence could potentially lead to an elevation of exogenous estrogen E2 levels and consequently the detrimental effects on $\dot{V}$ O_2peak_ decrease. Significantly lower absolute and relative $\dot{V}$ O_2peak_ values in CONS in the present work are suggested to be attributed to notably lower (−30% in CONS) E2 levels. However, as E2 levels are not significantly different and the potential effect of still remaining exogenous hormones from OCP intake is unclear, this interpretation has to be done with caution. Anyway, a possible reason for a lower $\dot{V}$ O_2peak_ in CONS might be a negative influence of E2 on the efficiency of the mitochondrion and, therefore, a reduced use of oxygen in the aerobic metabolism.

Interestingly, no changes between OCC phases have been found for submaximal $\dot{V}$ O_2_ and running economy under steady-state conditions. A higher maximal response in $\dot{V}$ O_2_ is suggested with an enhanced efficiency of the mitochondrion. However, this was not the case in the present work for submaximal $\dot{V}$ O_2_. Moreover, present results suggest that OCC phases do not seem to influence other factors determining running economy, like muscle stiffness, neuromuscular control, or other biomechanical factors. No changes in the speed associated with VT1 and VT2 were found, and therefore, it is suggested that the OCC phase does not influence threshold speeds. Consequently, the intensities that, on the one hand, elicit changes in [BLa^−^] and blood pH and, on the other hand, demarcate the boundary between steady-state and non-steady-state conditions are not influenced by OCP use.

In accordance with the previous literature, significantly higher values in CONS have been found in $\dot{V}$ E/ $\dot{V}$ O_2_ (Rechichi et al., 2008; Barba-Moreno et al., 2019) and $\dot{V}$ E/ $\dot{V}$ CO_2_ (Barba-Moreno et al., 2019) during submaximal exercise. However, the differences in $\dot{V}$ E (Rechichi et al., 2008; Barba-Moreno et al., 2019) have not been reproduced in the present study. Nevertheless, our findings confirm the notion of previous work (Rechichi et al., 2008; Barba-Moreno et al., 2019) that ventilatory inefficiency and a lower aerobic capacity during CONS are evident. Consequently, during the intake phase, an increase in respiratory drive is obvious. This is also reflected by a lower $\dot{V}$ O_2peak_ during CONS.

### Hormones

No significant differences between OCC phases have been found for hormonal analyses. This is in accordance with previous studies (Vaiksaar et al., 2011b; Jurimae et al., 2011), which might be explained by the suppressing effect of OCPs on endogenous hormones. On the other hand, previous research has shown lower E2 levels at the end of CONS compared to WITH. There was even a difference between early and late WITH, which leads to the suggestion that testing on days 2 or 3 in the present study might have been too early to detect a difference in E2 and, consequently, an effect on the mitochondrion or the aerobic system in general. The results of the present study show a very low level of E2; in some participants, it has even been below the detection limit of the analysis. This was probably due to the suppression of OCP intake and might also be explained by the timing of testing, which was in the early phase of WITH.

### Energy metabolism

In accordance with the total energy expenditure, no shift in substrate metabolism at 2.5 m∙s^−1^ was found. However, Mattu et al. (2020) and Giacomoni and Falgairette (2000) found significant differences in RER, with the latter also observing differences in CHO and fat oxidation and total energy expenditure, which were not observed in this study. The results have shown that different E2 levels are usually linked to changes in fat and CHO oxidation, with enhanced fat oxidation at high levels of E2 and consequently a similar change in the respiratory exchange ratio (Giacomoni and Falgairette, 2000). However, our results did not demonstrate these shifts and are in accordance with Vaiksaar et al. (2011b). These authors maintained that the disaccording findings of previous works might be due to differences in exercise modes (e.g., cycling vs. running vs. rowing), the duration and intensity of the exercise, and aerobic endurance level of the subjects. Present results suggest that at lower intensities in the heavy exercise intensity domain, no effects of OCP intake concerning energy expenditure are evident. The reduced ventilatory efficiency and potentially lower aerobic capacity do not affect energy expenditure. Furthermore, a ∼6% decrease in fat oxidation was found, which might be explained by a possible lower E2 concentration in CONS. This would be in accordance with recent works suggesting that a lower concentration reduces the oxidation of lipids (Giacomoni and Falgairette, 2000); however, these differences did not reach statistical significance. Based on the current results, we suggest that a greater difference in E2 between OCC phases might result in significant differences. Interestingly, Mattu et al. (2020) found no significant differences between OCC phases in time to exhaustion during a cycle-ergometer test, which suggests no influence of OCP intake on fatigue resistance.

### Cardiometabolic system

Previous literature found significantly higher [BLa^−^] during CONS than during WITH (Rechichi et al., 2008). However, Rechichi et al. (2008) stated as an explanation that the reported statistically significant differences of approximately 1 mmol.L^−1^ in [BLa^−^] are within the typical error of the measurement and might be caused by this error. In line with this statement, the present study found neither baseline nor maximal [BLa^−^] differences between OCC phases. Similar to [BLa^−^], no notable differences were demonstrated between OCC phases for submaximal and maximal HR. This suggests no differences in glycolytic metabolism and no influence on athletic performance concerning the cardiac system due to the intake of OCPs–when HR is suggested as a surrogate for the cardiac system.

### Anthropometry

Body mass did not differ significantly between OCC phases, which is in accordance with previous results for moderately trained (Giacomoni et al., 2000; Vaiksaar et al., 2011a; 2011b; Joyce et al., 2013) and endurance-trained athletes (Rechichi et al., 2008; Barba-Moreno et al., 2019). It is, therefore, suggested that neither the exogenous hormone intake nor fluctuations in the endogenous hormone profile had a notable influence on body mass.

### Subgroup analyses

A recent review by Elliott-Sale et al. (2020) suggested that the reasons for controversial findings might be explained by different concentrations of endogenous sex hormones, which vary individually through OCC phases and especially increase in the WITH phase. Moreover, physiologically meaningful differences in endogenous sex hormones have the potential to influence various physiological systems (e.g., Elliott-Sale et al., 2020; McNulty et al., 2020). Consequently, we analyzed the individual responses of E2, prog, and testo. Subsequently, we divided the total sample into subgroups where the inclusion criteria were lower hormone levels in CONS compared to WITH.

These further analyses showed influences on performance parameters when prog was increased (i.e., WITH) since [BLa^−^] at rest and maximal [BLa^−^] were significantly higher, represented by a *large* effect size and a significantly higher $\dot{V}$ O_2peak_ similar to the total sample. The higher [BLa^−^] might be associated with a slightly affected mitochondrial function, as previously demonstrated in patients with multiple sclerosis (Paling et al., 2011) or cancer (Vyas et al., 2016). However, this is in contrast to the decrease in $\dot{V}$ O_2max_ in the present work during CONS, which is related to decreased mitochondrial function and thus lower aerobic capabilities. It might be suggested that elevated body temperatures caused by higher prog levels have an influence on anaerobic glycolysis, but this remains unclear and warrants further investigation. In subgroups with increased E2 and testo, no statistically significant effects on performance parameters have been found. Although hormone levels of E2, prog, and testo were significantly higher in WITH of the subgroup, hormone fluctuation might not have been intense enough to influence performance and reach statistical significance.

### Limitations

A note of caution is due here since the fluctuation of endogenous blood hormones during daytime was not considered. Elliott-Sale et al. (2020) indicated that exogenous blood values might peak within 1 h after intake, which was not considered in the blood sample analysis. In future study designs, the measurement of hormonal-binding proteins (SHBG) should be considered to allow further interpretation. A potential cross reactivity between E2, prog, and testo assays and the components of the OCP might have influenced the analyses of hormone levels in venous blood samples. These cross reactions have been found to influence the analysis in previous animal studies, but the extrapolation of human data is unclear (Krasowski et al., 2014). In order not to influence participants’ daily routine and not to counteract the instructions for OCP intake, participants were advised to keep their usual OCP intake timing. A limitation of this study is that a detailed analysis of exogenous hormonal blood values is missing. It might have been of further interest to find out whether in WITH, exogenous hormones still “exist” in athletes’ bodies. Another limitation might be that submaximal parameters were obtained from a 1-min GXT protocol, which could affect the results derived from $\dot{V}$ O_2_ and $\dot{V}$ CO_2_ data due to different $\dot{V}$ O_2_ on-kinetics.

## Conclusion

Only small changes in some endurance parameters have been found. Interpretation has to be done with caution, as no differences in hormone levels have been found, but further analyses suggest that an increase in prog might be responsible for a lower efficient metabolism in the WITH phase. Although it seems that the effects might be negligible for most participants, in elite sports, even small physiological changes can differentiate between winning and losing. Therefore, individual analysis of hormonal influences seems to be justified in elite athletes. Hormonal contraception containing only synthetic prog (and no estrogen) might have fewer suppressing effects on hormonal levels. Consequently, endurance performance might be influenced differently, but research and data concerning this topic are lacking. As a future perspective, it might be useful to study participants using OCPs containing only progestogen, as well as the use of hormonal IUDs (similarly containing only synthetic prog).

## Data Availability

The raw data supporting the conclusion of this article will be made available by the authors, without undue reservation.

## Abbreviations

[BLa^−^], blood lactate; CONS, consumption phase; CHO, carbohydrate; CO_2_, carbon dioxide; E2, estradiol; GXT, graded exercise test; HR, heart rate; MAS, maximum aerobic speed; OCC, oral contraceptive cycle; OCP, oral contraceptive pill; O_2_, oxygen; prog, progesterone; SD, standard deviation; SEM, standard error of the mean; testo, testosterone; TTL, total energy expenditure; $\dot{V}$ E, minute ventilation; VT, ventilatory threshold; WITH, withdrawal phase.
